# How Ohio public library systems respond to opioid-related substance use: a descriptive analysis of survey results

**DOI:** 10.1186/s12889-024-18799-x

**Published:** 2024-05-17

**Authors:** Patrick M. Schnell, Ruochen Zhao, Sydney Schoenbeck, Kaleigh Niles, Sarah R. MacEwan, Martin Fried, Janet E. Childerhose

**Affiliations:** 1 Division of Biostatistics, College of Public Health, The Ohio State University, Columbus, OH USA; 2 Department of Research Administration, Summa Health, Akron, OH USA; 3 Recruitment, Intervention, and Survey Shared Resource, James Comprehensive Cancer Center, The Ohio State University College of Medicine, Columbus, OH USA; 4 Department of Internal Medicine, The Ohio State University College of Medicine, Columbus, OH USA; 5 Center for the Advancement of Team Science, Analytics, and Systems Thinking, The Ohio State University College of Medicine, Columbus, OH USA; 6 Department of Biomedical Education and Anatomy, The Ohio State University College of Medicine, Columbus, OH USA

**Keywords:** Public libraries, Opioid epidemic, Harm reduction, Naloxone, Ohio, Mixed-methods public health, Bioethics

## Abstract

**Background:**

Public libraries in the United States have experienced increases in opioid-related substance use in their communities and on their premises. This includes fatal and non-fatal overdose events. Some libraries have adopted response measures in their branches to deter substance use or prevent overdose. A small number of libraries around the nation have decided to stock the opioid antagonist naloxone (Narcan) for staff to administer to patrons who experience overdose. This response measure has generated extensive media attention. Although Ohio ranks fourth in age-adjusted drug mortality rate in the United States, there has been no investigation of whether Ohio libraries are observing opioid-related transactions, consumption, and/or overdose events, or which measures they have adopted in response to these activities. We conducted a multimethod survey with Ohio public library directors to identify the response measures they have adopted. We present descriptive findings from the quantitative and qualitative items in our survey.

**Methods:**

We conducted a cross-sectional 54-item multimethod survey of public library system directors (one per system) in Ohio. Directors of each of Ohio’s public library systems were invited to participate via email.

**Results:**

Of 251 library systems, 56 responded (22.3% response rate), with 34 respondents (60.7%) indicating awareness of opioid-related transactions, consumption, and/or overdose on their premises. Most (*n* = 43, 76.8%) did not stock naloxone in their buildings. Over half (*n* = 34, 60.7%) reported implementing one or more non-naloxone response measures. These measures focus on improving security for staff and patrons, deterring opioid-related transactions (purchases and exchanges) and consumption, and providing educational events on substance use. Nearly half (*n* = 25, 47.2%) partner with community organizations to provide opioid response measures. A similar proportion reported adequate funding to respond to opioid-related substance use (*n* = 23, 45.1%), and most (*n* = 38, 74.5%) reported adequate support from their boards and communities. Few respondents have implemented evaluations of their response measures.

**Conclusions:**

Ohio public libraries are responding to evidence of opioid-related transactions, consumption, and/or overdose on their premises with a range of measures that focus on substance use prevention and deterrence. Most Ohio library systems do not stock naloxone. Respondents indicated they prefer to call 911 and let first responders handle overdose events. The majority of respondents indicated their library systems have political capacity to respond to evidence of opioid-related substance use on their premises, but have limited operational and functional capacity. Findings suggest the need to revisit assumptions that public libraries are willing to stock naloxone to respond to overdose events, and that libraries have the resources to respond robustly to opioid-related transactions, consumption, and/or overdose on their premises.

**Supplementary Information:**

The online version contains supplementary material available at 10.1186/s12889-024-18799-x.

## Background

The United States (US) is undergoing a national opioid crisis. Drug overdose is currently the leading cause of accidental death in the US [1]. Between 1999 and 2021, nearly 645,000 people experienced a fatal opioid-related overdose (heroin, oxycodone, morphine, or fentanyl and its analogs) [2]. Since 2015, consumption of potent synthetic opioids such as fentanyl and carfentanil has been the leading cause of drug overdose fatalities [1]. In 2021, fentanyl accounted for 66% of 107,622 fatal overdoses in the US [3]. The COVID-19 pandemic has exacerbated opioid consumption across the US [4, 5].

There are more than 17,000 public libraries in the US. They offer vital community services and resources including restrooms, quiet spaces, privacy, and safety without the requirement or expectation of commerce. Public libraries are inclusive public institutions, welcoming those who are experiencing low income, homelessness, mental illness, or other health challenges. These features, which make such libraries valuable public institutions and community centers, may also make them attractive to persons who use substances including opioids or substances containing opioids.

Professional library organizations have responded with educational events and guidance documents [6–12]. Some library directors have decided to stock the opioid antagonist naloxone (Narcan), which can reverse respiratory depression caused by opioid overdose within minutes, and train their staff to administer it [13, 14]. The rapid reversal of respiratory distress by naloxone may be an important consideration for libraries located in rural or remote areas, which often have longer wait times for first responders than libraries located in urban and suburban areas. While stocking naloxone in libraries has received substantial media attention and commentary [15–18], libraries have also adopted a wide range of non-naloxone response measures, such as hiring additional security or social workers, installing security cameras, re-arranging furniture to improve staff surveillance of patron activity, installing syringe disposal bags, and holding educational events [19–21].

The idea that public libraries might be well-positioned to prevent overdose deaths on their premises by stocking naloxone and training staff to administer has received extensive attention. In 2017, the Lifesaving Librarians Act (H.R. 4259) proposed to provide funds for public libraries to buy Narcan rescue kits and train staff [22]. In 2018, Emergent Biosolutions, one of the manufacturers of Narcan, announced it would provide two free doses of the nasal spray to every public library and YMCA in the US [18, 23]. While the proposed bill did not pass, and most public libraries did not take up the Emergent Biosolutions offer, there has been substantial media attention to libraries experiencing and managing overdose on their premises [15–17]. In addition, the gray literature from the professional public library community depicts public libraries as choosing to be part of a national response to the epidemic along with law enforcement, healthcare facilities, and community services [7–11]. At the same time, some have criticized this narrative of public libraries stepping up to help manage a national opioid epidemic for ignoring the capacity of libraries to respond to the opioid crisis and potentially assigning additional duties to library staff, who have not trained to be emergency responders or clinical providers who manage substance use [18, 24, 25].

A growing body of survey and qualitative research conducted by teams in 15 states (Colorado, Connecticut, Florida, Illinois, Louisiana, Michigan, New York, North Carolina, Ohio, Pennsylvania, Rhode Island, Tennessee, Utah, Virginia, and Washington) provides insight into how some libraries have responded to substance use on their premises and communities and how the opioid epidemic has impacted their operations and programming, while highlighting diverse attitudes that public library professionals hold towards incorporating opioid response into their roles and work. A 2017 survey of 621 Pennsylvania library directors found that 12% of respondents had witnessed a drug overdose in the previous year. Libraries in this study had adopted two opioid response measures: stocking naloxone and training library employees to use it, and limiting patron time in restrooms to discourage injection drug use [26]. A qualitative study conducted with 20 directors of North Carolina public libraries found that nearly half of participants reported their library systems or branches had been directly affected by the crisis, but only a small proportion have trained staff to respond to on-site overdoses, established formal policies on opioid use, or stocked naloxone (25%, 15%, and 10% respectively) [27]. A cross-sectional survey of 356 library staff in five states (Colorado, Connecticut, Florida, Michigan, and Virginia) found that 12% of respondents had encountered at least one on-site overdose in the previous year and 0–33% of libraries in one state (Colorado) practiced naloxone stocking [28]. Other practices that libraries in this study adopted include installing syringe disposal containers and partnering with social workers and educators.

Two qualitative studies suggest that library personnel hold divergent, even polarizing, attitudes towards policing opioid-related transactions, consumption, and/or overdose on their premises, and using naloxone to reverse patron overdose. One is a two-part study that documents the decision-making processes shaping the responses of seven US public libraries in seven states (including one library in Ohio) to the opioid crisis [20, 21]. The other is a qualitative study of 44 public library staff attending a national meeting in 2018 [29]. Participants in both studies compared naloxone to cardiopulmonary resuscitation (CPR) but characterized it as safer than CPR, suggesting that some library staff hold a pragmatic view on using naloxone to rescue patrons from overdose. Yet staff in the 2018 study also characterized libraries as “de facto” safe injection sites. This characterization suggests that some staff believe libraries are not only ineffectively managing opioid-related substance use on their premises but are also inappropriately expanding the scope of their mission by managing overdose. Participants in both studies indicated they lack resources or institutional support to administer naloxone, are uncomfortable stepping into the role of first responder, and are concerned about personal harms and legal liability.

The divergent attitudes of library staff towards libraries managing substance use and overdose, along with the range of opioid response measures adopted by libraries indicated by this research suggest there is value in investigating how libraries in states with high rates of fatal opioid-related overdose are responding to opioid-related transactions, consumption, and overdose. Despite the high opioid-related overdose death rate in Ohio, to date there has been no single-study investigation of whether Ohio public libraries are observing or seeing evidence on their premises of [1] opioid-related transactions (e.g., purchases by, or exchanges between, patrons of illicit substances, including opioids or substances that have opioids added to them) [2], opioid consumption (e.g., injection using syringes and needles of prepared heroin and/or fentanyl or methamphetamine with fentanyl added, or oral consumption of prescription opioid medications such as oxycodone, whether obtained from a provider or purchased on the street), or [3] opioid-related overdose with respiratory distress or arrest. Nor has there been investigation of how Ohio public libraries are responding to observations or evidence of any of these phenomena. Ohio has consistently experienced some of the highest rates of opioid-related fatalities in the US, which suggests suitability for investigating public library responses. In 2020, it ranked as the fourth-highest state in rate of fatal drug overdose [30]. Prior to 2020, it had the second-highest age-adjusted opioid-related overdose death rate in the nation for two consecutive years (32.9 deaths per 100,000 people in 2017, increasing to 35.9 deaths per 100,000 in 2018) [31].

Additionally, there is an overarching gap in the published research, with no investigation in any state or nationally, of the *capacity* of public libraries to respond to opioid-related substance use and overdose on their premises. By “capacity,” we mean [1] operational capacity (whether public libraries have the funding, staffing, operational hours, location, and community partnerships to respond to opioid-related transactions, consumption, and/or overdose); [2] political capacity (whether library directors have the support of their boards, staff, and patrons to respond to opioid-related transactions, consumption, and/or overdose); and [3] functional capacity (whether responding to opioid-related transactions, consumption, and/or overdose falls within the mission of the public library and its role as a community space for all).

We designed a multimethod, multi-phase study with three aims to investigate whether Ohio public libraries are observing opioid-related transactions, consumption, and/or overdose on their premises and what response measures they are adopting (Aim 1); considerations (ethical, resource, political, and logistical) that shape directors’ decision-making to respond to these activities on their premises and in their communities (Aim 2); and directors’ perceptions of the duties of public libraries to manage a national opioid crisis and observations of how the epidemic may be transforming Ohio public libraries (Aim 3). In this article, we report findings from Aim 1, along with findings on stocking and administering naloxone as a response measure from Aim 2. We report the results of Aim 3 elsewhere.

## Methods

### Study design

The study, entitled, “Is Narcan the New CPR? The Capacity of Ohio Public Libraries as Opioid Responders,” was approved as exempt research (category #2c) by The Ohio State University Institutional Review Board in October 2021 (Study 2021E1077). For Aims 1 and 2 of this broader study, we conducted a cross-sectional, multimethod, REDCap [32, 33] survey of public library system directors (one per system) in Ohio [34]. The survey was administered online and presented 54 items that were mostly quantitative but included 18 open-ended questions soliciting write-in responses. We focus in this article on a descriptive analysis of the quantitative survey responses supplemented by qualitative analysis of open-ended survey responses on naloxone as a response measure.

### Survey design and administration

Our team developed a cross-sectional online multimethod survey using REDCap (Research Electronic Data Capture). REDCap is a secure, web-based software platform designed to support data capture for research studies, providing (1) an intuitive interface for validated data capture; (2) audit trails for tracking data manipulation and export procedures; (3) automated export procedures for seamless data downloads to common statistical packages; and (4) procedures for data integration and interoperability with external sources.

Study data were collected and managed using REDCap electronic data capture tools hosted at The Ohio State University. The 54-item survey asked questions in six domains: [1] awareness of two opioid educational and policy events in 2018 hosted by national and Ohio library organizations [2], observation or evidence of opioid-related transactions, consumption, and/or overdose at library system locations from 2017 to 2021 [3], implementation of naloxone opioid response measures in their system [4], implementation of non-naloxone opioid response measures [5], resource and support needs, and [6] impact of COVID-19 pandemic on their capacity to respond to opioid-related transactions, consumption, and/or overdose. The set of non-naloxone opioid response measures listed as choices in the survey was drawn from three sources: published investigations of public library responses to opioid-related transactions, consumption, and/or overdose [7, 8, 10, 13, 19–21, 27]; resource documents published by OCLC [6, 9]; and media reports of library responses to opioid-related transactions, consumption, and/or overdose [15–17]. At survey close, we solicited respondent interest in participating in a subsequent one-on-one interview and asked them to identify their role within their library system.

We identified library directors as our desired respondents for three reasons. Directors set the policies in their branches and are the primary decision-making authorities. They are familiar with their communities and patrons, and often have longer tenure with specific library systems than staff, who have high turnover. For example, they recognize regular patrons and talk to them. They also see changes to their communities over time. For these reasons, we identified them as the respondents that could provide the most informed responses to our questions. A second reason is that our advisory board of library directors, library science faculty, and leaders of state and national library professional organizations recommended that we survey directors. They reminded us that library staff experience survey fatigue and have been taxed by the Covid-19 pandemic. A third reason was pragmatic. Contact information for all library directors in Ohio is available to the public through state library organizations.

Our survey population included all 251 Ohio public library systems. These systems collectively have 481 branch locations, for a total of 732 library outlets. In May 2021, we retrieved an Excel spreadsheet of publicly available information on Ohio library systems from the State Library of Ohio website. This spreadsheet included director names and contact information. We used this resource to build our REDCap survey distribution mailout list. In November 2021, the State Librarian emailed a letter to all Ohio library directors describing the purpose of our survey and encouraging all to complete it. Using the director names and email addresses extracted from the State Library of Ohio spreadsheet, the REDCap system emailed a unique survey hyperlink to the director of each system on November 29, 2021. Five survey invitations returned to the study email account as undeliverable. The PI contacted the Deputy Director of the State Library of Ohio by email to correctly identify the email addresses for those directors and the team re-sent the unique survey invitation link to each using REDCap. The REDCap system sent two subsequent reminder emails to each respondent 7 and 14 days after the initial email (on December 6 and 13, 2021).

Additional information on each library system was linked from the 2019 Institute of Museum and Library Services (IMLS) Public Library Survey (PLS), an annual voluntary census of public libraries [35]. Relevant data from the PLS included National Center for Education Statistics (NCES) locale codes (rural, town, suburban, city), number of central and branch libraries, total staff count, and income sources and amounts. These four locale codes, each with three subtypes, were developed by the NCES in collaboration with the Bureau of Census [36]. The IMLS and PLS use these local codes to classify all US public libraries. The NCES defines **Rural** as a “Census-defined rural territory” ranging from less than 5 miles to more than 25 miles from an urbanized area and ranging from less than 2.5 miles to more than 10 miles to an urban cluster; **Town** as a “Territory inside an Urban Cluster” ranging from less than 10 miles to more than 35 miles from an “Urbanized Area;” **Suburban** as a “Territory outside a Principal City and inside an Urbanized Area” with a population of less than 100,000 to 250,000 or more; and **City** as a “Territory inside an urbanized area and inside a principal city” with a population of less than 100,000 to 250,000 or more [36].

### Data analysis

All quantitative survey data were analyzed using R v.4.1.2 [37]. For descriptive summaries, we reported medians and ranges for numeric variables and counts and frequencies for categorical variables. We also examined associations between categorical variables such as locale code and evidence of opioid use via Fisher’s exact test, and pairwise differences in numeric variables such as system income via the nonparametric Mann-Whitney U test.

We tabulated PLS data by level of survey response (no response, completed consent but entered no data on survey, entered data on survey). For each summary and statistical test, respondents were included if they responded to all relevant questions, regardless of response or non-response to any other survey question.

To understand respondents’ views on stocking naloxone in library systems and attitudes towards responding to opioid-related substance use, two staff with the Recruitment, Intervention, and Survey Shared Resource (RISSR) at the Ohio State University Comprehensive Cancer Center reviewed both complete and partially complete survey responses. They conducted qualitative and quantitative data analysis of all responses (yes/no and open-ended text) to two survey questions: [1] “Do any of your outlets stock naloxone?” and [2] “Do you believe that public libraries should respond to opioid activity on their premises?” The RISSR team focussed their analysis on open-text responses that respondents provided to explain their yes/no answers to these two survey questions. Using a small sample of surveys returned to the team, the team performed inductive coding on the open-ended text responses to these two questions to develop a list of themes. They reached agreement and developed a codebook using NVivo Plus qualitative software (Version 12.1 for Windows) [38]. After survey close, staff coded all additional survey responses using the codebook.

**Table 1:** Characteristics of library systems that did not and did respond to the survey

	Did not respond (*N* = 195)	Responded (*N* = 56)	OVERALL (*N* = 251)	*p*-value
NCES locale code				0.99
Rural	46 (23.6%)	12 (21.4%)	58 (23.1%)	
Town	82 (42.1%)	25 (44.6%)	107 (42.6%)	
Suburban	55 (28.2%)	16 (28.6%)	71 (28.3%)	
City	12 (6.2%)	3 (5.4%)	15 (6.0%)	
Branches in system				0.15
Mean (SD)	2.77 (4.62)	3.18 (4.16)	2.86 (4.51)	
Median [Min, Max]	1.00 [1.00, 41.0]	1.00 [1.00, 23.0]	1.00 [1.00, 41.0]	
Total staff (FTE)				0.03
Mean (SD)	33.9 (80.9)	45.0 (93.3)	36.4 (83.8)	
Median [Min, Max]	12.5 [0.880, 668]	21.4 [1.86, 641]	13.9 [0.880, 668]	
Total income (1000 USD)				0.07
Mean (SD)	3080 (8230)	4650 (11,800)	3430 (9140)	
Median [Min, Max]	934 [64.8, 65,500]	1390 [96.1, 77,900]	1030 [64.8, 77,900]	

All characteristics as reported by Institute of Museum and Library Services (IMLS) survey (2019). Total staff is full time equivalent (FTE) paid staff

## Results

We received survey responses from 56 of 251 library systems (22.3% response rate). Of those that responded, 53 (94.6%) submitted final responses, and three (5.4%) answered some questions but did not complete all items. An additional six respondents, not included in the 56 responses, completed the study consent form but did not enter any responses to the survey.

Table 1 displays the NCES locale code and library system size measures by level of response. Of the 56 responding library systems, 12 (21.4%) were classified as rural, 25 (44.6%) town, 16 (28.6%) suburban, and 3 (5.4%) city. There was no statistically significant association between locale code and response (*p* = 0.99). There was no statistically significant difference in total number of outlets (central plus branch) among systems that responded (median 1, range 1 to 23) and those that did not (median 1, range 1 to 41) (*p* = 0.15). There was a statistically significant difference in total number of paid full time equivalent (FTE) staff between respondents (median 21.4, range 1.9 to 641) and non-respondents (median 12.5, range 0.9 to 668) (*p* = 0.03). The total system incomes were slightly higher among respondents (median 1,390,000 USD, range 96,000 to 77,900,000 USD) than non-respondents (median 934,000 USD, range 65,000 to 65,500 USD), but the difference was not statistically significant (*p* = 0.07).

Table 2 displays reports of evidence of opioid-related substance use activities overall and by locale code. Knowledge of any evidence of opioid-related substance use was reported by 34 (60.7%) respondents, with reports more common in city (3, 100%) and town (19, 76.0%) than in suburban (8, 50.0%) and rural (4, 33.3%) locale codes, a statistically significant association (*p* = 0.03). Among all respondents, the most commonly-reported evidence was discarded syringes or needles (*N* = 26, 46.4%), followed by drug exchanges or purchases (*N* = 19, 33.9%), overdose events (*N* = 14, 25%), injection drug use (*N* = 9, 16.1%), discarded pills, tablets, bottles, patches, or blister packs (*N* = 9, 16.1%), and oral consumption of opioids (*N* = 1, 1.8%). Other evidence was reported by 4 (7.1%) respondents. Locale code was statistically significantly associated with reports of discarded syringes or needles (*p* = 0.02), drug exchanges or purchases (*p* = 0.04), and overdose events (*p* = 0.01), all of which were most common in city systems and least common in rural systems.

**Table 2:** Reported evidence of opioid-related substance use at library system locations 2017–2021

Evidence type	Rural (*N* = 12)	Town (*N* = 25)	Suburban (*N* = 16)	City (*N* = 3)	OVERALL (*N* = 56)	*p*-value	
Any	4 (33.3%)	19 (76%)	8 (50%)	3 (100%)	34 (60.7%)	0.027	
Discarded syringes or needles	2 (16.7%)	15 (60%)	6 (37.5%)	3 (100%)	26 (46.4%)	0.015	
Drug exchanges or purchases	1 (8.3%)	12 (48%)	4 (25%)	2 (66.7%)	19 (33.9%)	0.039	
Overdose events	0 (0%)	6 (24%)	5 (31.2%)	3 (100%)	14 (25%)	0.005	
Injection drug use	0 (0%)	4 (16%)	4 (25%)	1 (33.3%)	9 (16.1%)	0.199	
Discarded pills, tablets, etc.	1 (8.3%)	7 (28%)	1 (6.2%)	0 (0%)	9 (16.1%)	0.246	
Oral consumption of opioids	0 (0%)	1 (4%)	0 (0%)	0 (0%)	1 (1.8%)	1.000	
Other	2 (16.7%)	1 (4%)	1 (6.2%)	0 (0%)	4 (7.1%)	0.472	

A strong majority (*N* = 47, 83.9%) reported that they believe that public libraries should respond to opioid-related transactions, consumption, and/or overdose on their premises. One respondent wrote, “We need to keep libraries safe for all ages. Any illegal activity has to be addressed to maintain safety.” Other respondents highlighted the importance of individual well-being, regardless of the cause of the problem, and emphasized that libraries are inclusive spaces. Providing assistance is a central tenet of public libraries, respondents wrote, and libraries would be expected to include responses to substance use. One respondent wrote, “Libraries can help save lives. People need real help and they trust libraries. We should be part of the solution.” Some respondents indicated that responses to substance use were necessary to preserve community perceptions of the library overall. For example, one respondent wrote, “I think if a library does nothing with regard to opioid activity on their premises they will lose the general public’s support for having a library in the first place,” while another said, “A fatality on the premises could be linked to or perceived as neglect on the library’s part.” Respondents also expressed concerns about the internal resources required to respond to opioid-related substance use. One respondent worried about the dangers of exposing staff to substance use and overdose.

Table 3 displays opioid response measures by locale code. Naloxone was stocked by 13 (23.2%) systems and was most common in city systems (*N* = 2, 66.7%), followed by suburban (*N* = 5, 31.3%), town (*N* = 6, 24.0%), and rural (*N* = 0, 0%) systems. Stocking of naloxone was statistically significantly associated with locale (*p* = 0.046). Three respondents (5.4%) reported evaluating the response measures used (collecting statistics; talking to staff or soliciting staff feedback and reviewing bathroom usage; and using data from postcard mailers attached to Deterra drug disposal bags).

**Table 3:** Reported opioid response measures adopted by library systems

Response type	Rural (*N* = 12)	Town (*N* = 25)	Suburban (*N* = 16)	City (*N* = 3)	OVERALL (*N* = 56)	*p*-value
Stocking naloxone	0 (0%)	6 (24%)	5 (31.2%)	2 (66.7%)	13 (23.2%)	0.046
Any non-naloxone	5 (41.7%)	14 (56%)	12 (75%)	3 (100%)	34 (60.7%)	0.174
Any facility	5 (41.7%)	12 (48%)	8 (50%)	2 (66.7%)	27 (48.2%)	0.930
Installing security cameras	3 (25%)	6 (24%)	5 (31.2%)	0 (0%)	14 (25%)	0.870
Restrooms by request only	4 (33.3%)	6 (24%)	3 (18.8%)	0 (0%)	13 (23.2%)	0.716
Moving desks or shelving to improve sightlines for staff	2 (16.7%)	4 (16%)	5 (31.2%)	2 (66.7%)	13 (23.2%)	0.192
Locking restrooms	1 (8.3%)	2 (8%)	0 (0%)	0 (0%)	3 (5.4%)	0.654
Limiting opening hours	0 (0%)	0 (0%)	0 (0%)	1 (33.3%)	1 (1.8%)	0.054
Installing blue lights in restrooms	0 (0%)	0 (0%)	0 (0%)	0 (0%)	0 (0%)	1.000
Any resources	0 (0%)	9 (36%)	9 (56.2%)	2 (66.7%)	20 (35.7%)	0.004
Providing Deterra drug disposal bags	0 (0%)	4 (16%)	4 (25%)	0 (0%)	8 (14.3%)	0.294
Holding opioid education events	0 (0%)	4 (16%)	4 (25%)	0 (0%)	8 (14.3%)	0.294
Hiring security guards to prevent substance sales or use	0 (0%)	2 (8%)	5 (31.2%)	0 (0%)	7 (12.5%)	0.073
Installing syringe disposal containers	0 (0%)	3 (12%)	2 (12.5%)	1 (33.3%)	6 (10.7%)	0.304
Making social workers available	0 (0%)	2 (8%)	0 (0%)	1 (33.3%)	3 (5.4%)	0.140
Making peer educators available	0 (0%)	1 (4%)	0 (0%)	0 (0%)	1 (1.8%)	1.000
Making nurses or nurse practitioners available	0 (0%)	0 (0%)	0 (0%)	0 (0%)	0 (0%)	1.000
Distributing fentanyl test strips	0 (0%)	0 (0%)	0 (0%)	0 (0%)	0 (0%)	1.000
Any referrals	0 (0%)	4 (16%)	4 (25%)	0 (0%)	8 (14.3%)	0.294
Referring to drug treatment	0 (0%)	4 (16%)	3 (18.8%)	0 (0%)	7 (12.5%)	0.421
Referring to naloxone distributors	0 (0%)	2 (8%)	1 (6.2%)	0 (0%)	3 (5.4%)	1.000
Referring to needle exchange sites	0 (0%)	1 (4%)	1 (6.2%)	0 (0%)	2 (3.6%)	1.000

**Table 4:** Proportions of library systems not observing and observing evidence of opioid-related activity that also stock naloxone

Evidence type	Proportion stocking naloxone		*p*-value
	No evidence	Evidence	
Any	2 of 22 (9.1%)	11 of 34 (32.4%)	0.056
Discarded syringes or needles	4 of 30 (13.3%)	9 of 26 (34.6%)	0.111
Drug exchanges or purchases	6 of 37 (16.2%)	7 of 19 (36.8%)	0.104
Overdose events	8 of 42 (19%)	5 of 14 (35.7%)	0.274
Injection drug use	9 of 47 (19.1%)	4 of 9 (44.4%)	0.189
Discarded pills, tablets, etc.	11 of 47 (23.4%)	2 of 9 (22.2%)	1.000
Oral consumption of opioids	13 of 55 (23.6%)	0 of 1 (0%)	1.000
Other	13 of 52 (25%)	0 of 4 (0%)	0.563

Most respondents indicated that they did not, or would not, stock naloxone to respond to patron overdose. Of the 43 responding systems not stocking naloxone, 17 (40%) cited availability of nearby resources such as police or EMS, 15 (35%) cited lack of need (e.g., no history of drug or overdose activity on premises), 8 (19%) cited no responsibility to intervene (e.g., not the staff’s job, staff declined training), 6 (14%) cited liability concerns, 6 (14%) cited lack of internal resources (e.g., training, staff), and 3 (7%) cited perceived danger.

Qualitative responses from respondents elaborate on these themes and indicate that stocking naloxone is a polarizing issue for library directors. Several respondents stated they relied on first responders to manage any emergency, including overdose events (“We have a local police department that is within one block and in our small town we think upon calling 911 they or EMS would be here within a couple of minutes”). Others characterized stocking naloxone as beyond the scope of services, “inappropriate,” or not a response measure they had considered. Among libraries that had contemplated stocking naloxone at some point, respondents identified lack of staff training to administer naloxone and to manage substance-related situations on the premises as barriers. They identified related concerns that asking staff to undergo naloxone training and administer naloxone might conflict with their values (e.g. “Staff members have varying beliefs on naloxone. I did not want them to feel pressured to go against their beliefs.”). Respondents indicated they were uncertain about whether staff held any responsibility to intervene in overdose and worried that providing overdose rescue might endanger staff or burden them with tasks beyond their role duties (e.g., “Many of our staff are not comfortable intervening in an overdose event, so we chose not to create that implicit requirement”).

Table 4 displays stocking of naloxone by evidence of opioid use. Although no associations were statistically significant (*p* = 0.056 to 1.0), naloxone was stocked by a greater proportion of library systems that reported any evidence of opioid-related transactions, consumption, and/or overdose (32.4%) than by those that did not (9.1%), and similarly for the following specific evidence types: drug exchanges or purchases (36.8% vs. 16.2%), injection drug use (44.4% vs. 19.1%), discarded syringes or needles (34.6% vs. 13.3%), and overdose events (35.7% vs. 19.0%). Naloxone was stocked by a lesser proportion of systems that did versus did not report oral consumption of opioids (0% vs. 23.6%), discarded pills, tablets, bottles, patches, or blister packs (22.2% vs. 23.4%), and other evidence (0% vs. 25%).

Non-naloxone measures adopted (Table 3) included facility changes (*N* = 27, 48.2%); most commonly, installing security cameras in outlets (*N* = 14, 25%), making restroom access available by request only (*N* = 13, 23.2%), and moving desks or shelving to improve sightlines for staff (*N* = 13, 23.2%). Locale was not statistically significantly associated with the implementation of facility changes overall (*p* = 0.9) nor with any specific change (*p* = 0.054 to 1.0). Adding resources (*N* = 20, 35.7%) was statistically significantly associated with locale (*p* = 0.004), most commonly for city systems (*N* = 2, 66.7%) and least commonly for rural systems (*N* = 0, 0%). Resources added most frequently included providing Deterra drug disposal bags (*N* = 8, 14.3%), holding opioid education events (*N* = 8, 14.3%), and hiring security guards to patrol for drug activity (*N* = 7, 12.5%). There were no statistically significant associations between specific resources and locale (*p* = 0.07 to 1.0). Making referrals (*N* = 8, 14.3%) was not statistically significantly associated with locale (*p* = 0.3), nor were specific referrals to drug treatment (*N* = 7, 12.5%, *p* = 0.4), naloxone distributors (*N* = 7, 5.4%, *p* = 1.0), or needle exchange sites (*N* = 2, 3.6%, *p* = 1.0).

**Table 5:** Reported aspects of capacity to respond to opioid-related substance use

Capacity to respond to opioid-related substance use	*N* (%)
Partnering with community organizations	25 (47.2%)
Adequate funding	23 (45.1%)
Adequate support from board, organizations, and community	38 (74.5%)
Impacted by COVID-19 pandemic	10 (18.9%)

Partnering with community organizations to respond to opioid-related transactions, consumption, and/or overdose was reported by 25 (47.2%) respondents (Table 5), adequate funding to respond, by 23 (45.1%), and adequate support from board, organizations, and community to respond, by 38 (74.5%). There were no statistically significant associations with locale (all *p* = 0.2).

The COVID-19 pandemic was reported to have affected the ability of 18.9% of library systems to respond to potential or actual opioid-related transactions, consumption, and/or overdose. Impact was most reported among city (50.0%) and suburban (26.7%) systems and least among town (20.0%) and rural (0.0%) systems. The association was not statistically significant (*p* = 0.2).

## Discussion

Evidence of opioid-related substance use (transactions, consumption, and/or overdose) is commonly observed in public library systems in Ohio. In our study, 16% of respondents reported injection drug use being observed at their library systems, and 25% reported overdose events. An earlier study of library systems in five states (Ohio excluded) found a wider range of rates of observed injection drug use (7–36%) and lower overdose rates (10–17%) [28]. However, our study presents responses regarding a wider range of specific types of evidence of opioid-related transactions, consumption, and/or overdose. Most commonly, this is discarded syringes or needles, and drug exchanges or purchases.

A low proportion of libraries report stocking naloxone (23%) as a response to opioid-related transactions, consumption, and/or overdose, and we show that naloxone stocking increases with urbanicity. Although the relationship was not statistically significant, naloxone was stocked by a higher proportion of systems reporting observing various types of evidence of opioid-related substance use (e.g., 32% of those reporting observing any evidence of opioid-related activity versus 9% of those who did not). A higher proportion of respondents made facility changes (48%) or provided resources (36%) in response to opioid-related substance use, and some provided referrals to support programs (14%). Although more than half of respondents reported adequate support from boards, organizations, and communities (75%), less than half reported adequate funding (45%).

Responses to manage opioid-related substance use on library premises may have unintended benefits and consequences. Some response measures (e.g., moving furniture and shelving) align with existing space management practices. These may be minimally disruptive to patrons or beneficial. Others (e.g., hiring security or healthcare personnel, holding educational events, making referrals) are expansive measures that add new resources to libraries. These measures may offer unanticipated benefits to patrons and communities. For example, social workers on premises can refer patrons to mental health services unrelated to substance use or promote resources that are unknown to library staff. Several measures (e.g., locking restrooms or making them available by request only; limiting opening hours) have the potential to negatively impact patrons by reducing access to library services. For example, in some communities, the library may be the only institution that has free restrooms open to the public. Locking or limiting restroom access may affect low-income and homeless populations that cannot use restrooms in retail settings.

Is naloxone the new CPR? This was the question we posed in our study title. One of the questions driving our study was whether Ohio library directors believe that their staff should be prepared to administer naloxone to patrons experiencing overdose. The idea that administering naloxone to someone experiencing opioid-related respiratory distress is “like” cardio-pulmonary resuscitation (CPR) is an appealing metaphor that participants in other studies and media commentators have used. It suggests that administering naloxone is easy to do, and that all citizens have a duty to administer naloxone to someone experiencing overdose, just as citizens had duties to undergo CPR training and rescue someone experiencing cardiovascular distress in previous decades. Our findings suggest caution in making these assumptions. Most survey respondents do not endorse stocking naloxone. Nor do they provide naloxone training for their staff or expect their staff to administer naloxone. It is worth noting that using naloxone remains stigmatized in ways that CPR administration arguably does not. Naloxone-related stigma may shape the reluctance of some libraries on this response measure.

We draw some preliminary conclusions from our findings about the capacity of Ohio public libraries to respond to opioid-related transactions, consumption, and/or overdose. Survey responses indicate that operational capacity of Ohio libraries to introduce opioid responses measures, such as hiring additional staff or introducing programming, is limited or non-existent. Even while respondents indicated their library systems have political capacity to adopt response measures, a majority of respondents reported inadequate funding, staffing shortages, and limited hours due to the Covid-19 pandemic. In terms of functional capacity, a strong majority of respondents indicated that libraries have the responsibility to respond to substance use and overdose on their premises. For example, a common theme in the open-ended survey responses was the importance of safeguarding libraries as a welcoming community space for all. However, those who responded to our questions on functional capacity drew clear lines around *how* to appropriately respond to overdose.

Notably, only three respondents (5.4%) that had adopted any opioid response measure also reported evaluating the impacts or effectiveness of that measure. Reasons are unclear. It is possible that library personnel may not perceive certain response measures (e.g., moving furniture or locking restrooms) as interventions. Other possible reasons include lack of professional training or experience in conducting evaluations amongst library personnel, and resource limitations (particularly staff time). Given that many response measures may have both positive and negative consequences for patrons and the community, library directors and researchers should consider evaluating measures to understand their effectiveness, as well as their related impacts on patrons and their communities.

The primary limitation of our study is the response rate of 22.3%. While this is low, it is comparable to the rates of other research teams conducting surveys with this population. For example, our rate is nearly identical to the response rate for a recent five-state survey of library staff on substance use and overdose [28]. Factors that may have contributed to the low response rate for this survey were [1] the short survey window (the survey was open for only three weeks) [2], the timing of survey open and close between two holiday periods (Thanksgiving and winter holiday break), and [3] the high number of surveys that public library directors and staff receive throughout the year. We note that only three responses were received from urban library systems, a response rate (20%) similar to the overall rate. As a result, differential propensity to respond to the survey depending on perceived urgency of the opioid use challenge and willingness to take mitigating measures may have a large effect on our conclusions. Although we framed our survey to emphasize opioid-related activity, it should be noted that not all injection use is illicit substance use nor opioid use. For example, insulin, a medication to manage diabetes, can be injected, as can crystal methamphetamine, an illicit stimulant. Finally, although we explored several facets of Ohio libraries’ responses, results may differ considerably in other states.

## Conclusions

Most directors that responded to our survey reported that they or their staff had observed, or found evidence of, opioid-related substance use transactions, consumption, and/or overdose on their premises. Most respondents have adopted one or more response measures in their systems intended to deter or prevent opioid-related substance use. However, while many respondents agreed that their library systems have political capacity to respond to opioid-related substance use, with broad support for implementing non-naloxone opioid response measures, most Ohio library systems lack operational and functional capacity to respond to the wide range of opioid-related substance use activities they report. Given this finding, it is difficult to agree with the notion that Ohio public libraries are equipped to be “opioid responders.” That is, they do not have the resources to manage the opioid epidemic in and through the public space of libraries. Many library systems may be prepared to respond to concerns about the safety and use of the space for all patrons, but most directors are unwilling for their staff to administer naloxone to patrons experiencing overdose. Library staff respond as they do for other emergencies: they call 911. This is a clear boundary for most library directors who responded to our survey, one that should be considered in the development of future public health and public library policy guidance.

This survey report provides granular descriptions of which types of library systems observe which types of evidence of opioid activity and implement which responses. Additional research might explore why directors select specific response measures to use in their systems, the effectiveness of response measures they adopt, and barriers to libraries evaluating the outcomes of response measures. These data would in turn inform an understanding of types of support, education, and policy changes needed to support libraries in managing and reducing opioid-related substance use on their premises, and how to support their patrons with appropriate resources. However, it is also important to investigate structural reasons why public library system employees may be placed in the de facto role of first responder and public health interventionist despite the lack of professional training and commensurate compensation, and whether the choice to respond at all is perceived as voluntary or necessary.

### Electronic supplementary material

Below is the link to the electronic supplementary material.

Supplementary Material 1

Supplementary Material 2

## Data Availability

The dataset supporting the conclusions of this article is included within the article and its additional files.

## Abbreviations

IMLS: Institute of Museum and Library Services
NCES: National Center for Education Statistics
PLS: Public Library Survey
