# Supplementary material for: How Ohio public library systems respond to opioid-related substance use: a descriptive analysis of survey results
Source: BMC Public Health. 2024 May 17;24:1336. doi: 10.1186/s12889-024-18799-x (PMC11101333; doi:10.1186/s12889-024-18799-x)
Supplement: Supplementary file 1 — Supplementary Material 1 [file 12889_2024_18799_MOESM1_ESM.docx]

**Supplement S1: Survey Instrument**

| \| The Capacity of Ohio Public Libraries as Opioid Responders \|  \| \| --- \| --- \|  Thank you for completing informed consent for this survey. You may now start the survey |
| --- | --- | --- |
| Awareness of library organization activities on opioid activity |
| This survey is building on previous work by public library organizations about how the opioid crisis is affecting public libraries. We want to tell you about some of this work.   In 2018, the Public Library Association, OCLC, and WebJunction ("PLA-OCLC / WebJunction") sponsored the project Public Libraries Respond to the Opioid Crisis with Their Communities with support from the Institute of Museum and Library Services.   The project produced [three resources](https://www.oclc.org/research/public-libraries-opioid-crisis.html) to help public libraries respond to the opioid crisis:   - A summary report describing the scope of the opioid problem and its impact on public libraries - Case studies outlining responses of individual community libraries to the problem - A "Call to Action" outlining strategies and resources to guide libraries   This was followed by the OhioNet-State Library of Ohio regional events Opioids in Communities--Libraries in Response in August 2018.  It has been three years since these two initiatives. Since then, the COVID-19 pandemic has impacted every library and community in Ohio. Opioid consumption and overdose rates have risen with the pandemic.  We would now like to ask you about your awareness of these initiatives and whether they have been helpful to you.   1. Were you aware of the “Public Libraries Respond to the Opioid Crisis with Their Communities” project?  - Yes - No  1. Have you used any of the resources from the “Public Libraries Respond to the Opioid Crisis with Their Communities” project?  - Yes - No  1. Were you aware of the "Opioids in Communities--Libraries in Response" cross-Ohio event held in August 2018?  - Yes: I organized, hosted, or attended one or more panels - Yes: I was aware of the event, but did not attend any of the panels - No  1. Is there anything else that you would like us to know about how public library organizations are responding to the opioid crisis? [write-in response space] |
| Opioid activity 2017-2021 |
| Next, we would like to ask about opioid activity at outlets in your library system. It may be hard to know whether drug activity is opioid activity. However, we are interested in knowing whether you have:   - observed what you believe is opioid activity at your outlets, or - found evidence of opioid activity at your outlets.   **Examples of activities**:   - Drug exchanges or purchases - Injection drug use - Oral consumption of opioids - Overdose events   **Examples of evidence:**   - Discarded syringes or needles - Discarded pills, tablets, bottles, patches, or blister packs  1. Thinking about the last five years (2017-2021), have you been aware of any opioid activity at your outlets?  - Yes - No [Skip to Q10]  1. What have you observed or found? Please check all that apply.  - Drug exchanges or purchases - Injection drug use - Discarded syringes or needles - Oral consumption of opioids - Discarded pills, tablets, bottles, patches, or blister packs - Overdose events - Other [write-in response space]  1. Does your library system keep records about opioid activity on your premises?  - Yes [Skip to Q8] - No [Skip to Q10]  1. About when did your library system start keeping records about opioid activity?   Month: [respondent can select month from menu] Year: [respondent can select year from menu]   1. Has the COVID-19 pandemic interrupted recording-keeping about opioid activity?  - Yes - No  1. Is there anything else that you would like us to know about opioid activity in your library system? [write-in response space] |
| Opioid response measures: Naloxone |
| Some library systems have decided to stock the opioid antagonist naloxone (Narcan) to rescue patrons or others who overdose after using opioids. Please answer these questions even if you have never observed opioid use or activity at your outlets.   1. Do you stock naloxone in any of your outlets?  - Yes - No [Skip to Q20]  1. About when did your library system start to stock naloxone?   Month: [respondent can select month from menu] Year: [respondent can select year from menu]   1. Have your staff had naloxone training?  - Yes - No  1. Have you or your staff ever used naloxone to rescue anyone on your premises from an opioid overdose?  - Yes - No  1. Who is authorized to use naloxone in your library system? Please check all that apply.  - Director / Assistant Director - Staff who have had naloxone training - Staff who have not had naloxone training - Patrons and bystanders  1. Who provides naloxone to your library system? Please check all that apply.  - Local public health department - Harm Reduction Ohio - Project DAWN - Local pharmacy - Other [write-in response space]  1. Have you ever had challenges in obtaining naloxone?  - Yes - No [Skip to Q21]  1. What challenges have you had in obtaining naloxone? [write-in response] 2. Have you ever had to purchase naloxone for your library system?  - Yes [Skip to Q21] - No [Skip to Q21]  1. Why has your library system decided not to stock naloxone? [write-in response] 2. Is there anything else that you would like us to know about stocking or using naloxone in your library system? [write-in response] |
| Opioid response measures: Non-naloxone |
| We would like to know about other opioid response measures that your library system may have adopted that are NOT related to naloxone.   1. Has your library system developed any written policies or procedures about opioid activity?   For example:   - Policies on how to respond to someone who appears to be buying, exchanging, or using drugs - Procedures for handling discarded syringes or medications - Yes - No  1. Has your library system provided staff training on how to respond to a suspected opioid overdose?  - Yes - No  1. Has your library system adopted any of the following non-naloxone response measures? Please check all that apply.   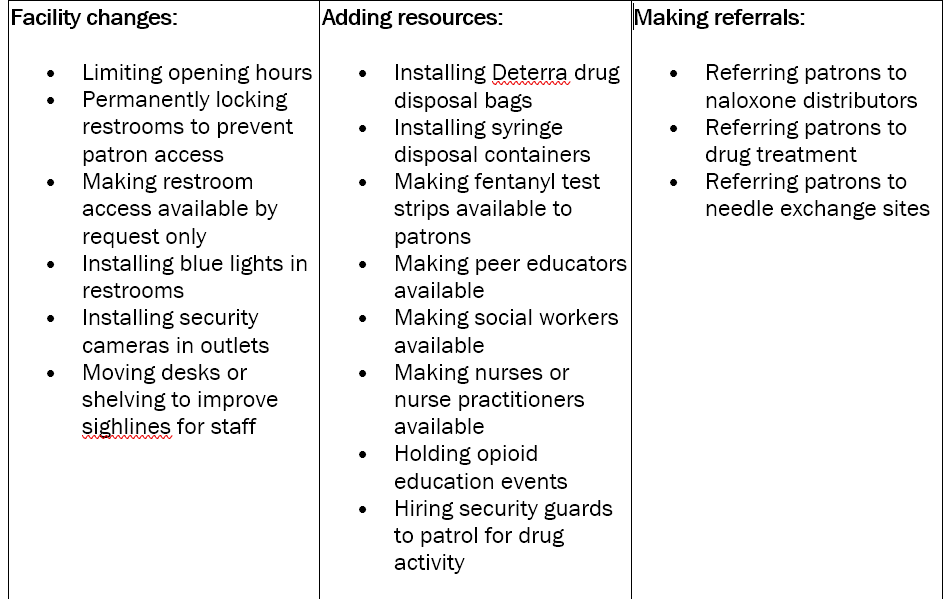   - Yes, our system has adopted at least one measure - No, our system has not adopted any measures [Skip to Q30] |
| 1. Which of the following non-naloxone response measures has your library system adopted? Please check all that apply.   **Facility changes:**   - Limiting opening hours - Permanently locking restrooms to prevent patron access - Making restroom access available by request only - Installing blue lights in restrooms - Installing security cameras in outlets - Moving desks or shelving to improve sightlines for staff   **Adding resources:**   - Installing Deterra drug disposal bags - Installing syringe disposal containers - Making fentanyl test strips available to patrons - Making peer educators available - Making social workers available - Making nurses or nurse practitioners available - Holding opioid education events - Hiring security guards to patrol for drug activity   **Making referrals:**   - Referring patrons to naloxone distributors - Referring patrons to drug treatment - Referring patrons to needle exchange sites   **Other (please describe)** [write-in response]   1. Has your library system evaluated whether these measures are having an impact?  - Yes - No [Skip to Q28]  1. How are you evaluating the impact of these measures? For example, have you surveyed your staff or collected statistics? [write-in response] 2. Going forward, if you could only adopt ONE measure, which one would you choose? [auto-populate from the selected responses to Q25, including “other” if that was selected] 3. Why would you choose this measure? [write-in response] 4. Is there anything else that you would like us to know about adopting non-naloxone opioid response measures in your library system? [write-in response] |
| Resource and support needs |
| We would like to know about the resources and supports that your library system needs to respond to **potential** or **actual** opioid activity. Please answer these questions even if you have not observed any opioid activity in your library system.   1. Does your library system partner or work with any community organizations to respond to opioid activity?  - Yes - No [Skip to Q33]  1. Which organizations? [write-in response] 2. Does your library system have adequate funding to respond to potential or actual opioid activity?  - Yes [Skip to Q36] - No  \|  \| \| --- \|  1. Did you have to find additional funds outside your budget to adopt any measures?  - Yes - No [Skip to Q36]  1. If your library system had adequate funding, how would you be able to respond to opioid activity? [write-in response] 2. Does your library system have adequate support from your board, organizations, and/or community to respond to potential or actual opioid activity?  - Yes [Skip to Q38] - No  1. If your library system had adequate support from your board, organizations, and/or community, how would you be able to respond to opioid activity? [write-in response] 2. What other resources does your library system need to respond to potential or actual opioid activity? [write-in response] 3. Do you believe that public libraries should respond to opioid activity on their premises?  - Yes - No  1. Please tell us why: [write-in response] 2. Is there anything else that you would like us to know about the capacity of your library system to respond to opioid activity? [write-in response] |
| Impact of COVID-19 pandemic |
| The COVID-19 pandemic has impacted every library in Ohio. Because we have also seen an increase in opioid activity and overdose rates in Ohio communities with the pandemic, we would like to ask you about how the pandemic has affected your library system and outlets.  Have any of your outlets been closed due to the Covid-19 pandemic?   - Yes - No [Skip to just above Q43]      1. What is the longest period of time that any of your outlets have been closed due to the Covid-19 pandemic? [respondent selects from drop-down menu with 13 choices: individual number of months (1 to 12) and greater than 12 months]   Thinking about when your library outlets have been OPEN since the start of the Covid-19 pandemic in March 2020:   1. As of today, do you have reduced hours at any outlet because of the COVID-19 pandemic?  - Yes - No  1. As of today, do you have fewer staff or reduced staff hours at any outlet because of the COVID-19 pandemic?  - Yes - No  1. Have you introduced any NEW programming or services at any outlets because of the COVID-19 pandemic?  - Yes - No [Skip to Q47]  1. What new programming or services have you introduced? [write-in response] 2. Has the COVID-19 pandemic affected the ability of your library system to respond to potential or actual opioid activity?  - Yes - No [Skip to Q49]  1. How has the COVID-19 pandemic affected the ability of your library system to respond? [write-in response] 2. Is there anything else that you would like us to know about how the Covid-19 pandemic has impacted the operation of your library system? [write-in response] |
| Interview recruitment |
| Part 2 of this study is an optional one-on-one interview by video or telephone. It will be scheduled in the Fall of 2021 or Winter of 2022. The interview is an opportunity for you to tell us more about how your library is responding to the opioid crisis and what roles public libraries are playing in managing the opioid crisis in the US.   1. Are you interested in participating in this interview?  - Yes [Prompts email to PI and study coordinator] - No [Skip to Q52]  1. To discuss the interview with you, do we have your consent to contact you at the email address that we used to send you this survey?  - Yes [Skip to Q52] - No. Please contact me at this email address: [write-in response] |
| Respondent demographics |
| 1. What is your current position in this library system? (You may have worked in other library systems. Please answer only for your current position.) [write-in response] 2. About when did you start in this position in this library system? (You may have worked in other library systems. Please answer only for your current position.)   Month: [respondent can select month from menu] Year: [respondent can select year from menu]   1. Is there anything else that you would like us to know about your role in this library system? [write-in response] |
| **Thank you very much for your time. Your responses to this survey are valuable. We are grateful that you took the time to answer.** |
